# The Utility of Peripheral Blood Leucocyte Ratios as Biomarkers in Neonatal Sepsis: A Systematic Review and Meta-Analysis

**DOI:** 10.3389/fped.2022.908362

**Published:** 2022-07-22

**Authors:** Juanjuan Zhang, Jun’an Zeng, Liangjuan Zhang, Xiping Yu, Jinzhen Guo, Zhankui Li

**Affiliations:** Department of Neonatology, Northwest Women’s and Children’s Hospital, Xi’an, China

**Keywords:** neutrophil-to-lymphocyte ratio, platelet-to-lymphocyte ratio, immature-to-total neutrophil ratio, immature-to-mature neutrophil ratio, neonatal sepsis, meta-analysis

## Abstract

**Background:**

Early stage diagnosis of neonatal sepsis (NS) remains a major roadblock due to non-specific symptoms and the absence of precise laboratory index tests. The full blood count is a relatively cheap, universal, and rapid diagnostic test.

**Method:**

This study assessed the diagnostic accuracies of immature-to-total neutrophil ratio (ITR), immature-to-mature neutrophil ratio (IMR), neutrophil-to-lymphocyte ratio (NLR), and platelet-to-lymphocyte ratio (PLR) used in the diagnosis of NS. Included studies were retrieved by searching four major databases and relevant references, and reviewed based on the inclusion/exclusion criteria. Pooled sensitivities and specificities were calculated, *I*^2^ was utilized to test for heterogeneity, and the source was investigated *via* meta-regression analysis.

**Results:**

Finally, 38 studies passed the eligibility criteria. A total of thirty-one studies (6,221 neonates) included data on the ITR, eight studies (1,230 neonates) included data on the IMR, seven studies (751 neonates) included data on the NLR, and two studies (283 neonates) included data on the PLR. The summary sensitivity estimates with 95% confidence interval (CI) for the ITR, IMR, NLR, and PLR tests were, respectively, 0.74 (95% CI: 0.66–0.80), 0.74 (95% CI: 0.54–0.88), 0.73 (95% CI: 0.68–0.78), and 0.81 (95% CI: 0.55–1.00). The summary specificity values for the ITR, IMR, NLR, and PLR tests were 0.83 (95% CI: 0.77–0.87), 0.89 (95% CI: 0.80–0.94), 0.69 (95% CI: 0.57–0.79), and 0.93 (95% CI: 0.81–1.00), respectively. The area under the summary receiver operating characteristic curves for the ITR, IMR, and NLR tests were 0.85 (95% CI: 0.82–0.88), 0.91 (95% CI: 0.88–0.93), and 0.75 (95% CI: 0.71–0.79). The PLR could not be evaluated because only two studies included pertinent data.

**Conclusion:**

The NLR test might not be sufficiently accurate in precisely diagnosing NS. The ITR and IMR tests alone can improve the accuracy of NS diagnosis, but the marked heterogeneity and the limited number of studies prevented us from reaching any definitive conclusions. Thus, further studies are warranted to validate these findings.

**Systematic Review Registration:**

[https://www.crd.york.ac.uk/prospero/], identifier [CRD42021247850].

## Introduction

Globally, sepsis is a serious and fatal health concern in neonates. Approximately 22/1,000 live births encounter neonatal sepsis (NS) with a mortality rate of 11–19% (1). NS symptoms could often be non-specific and can widely vary from mild and chronic to acute onset with rapid relapse. Moreover, certain diagnostic limitations of blood examinations collectively hinder the early diagnosis and management of NS. Notably, the major contributing factors to worsening outcomes in life-threatening NS are mostly the misdiagnosis and misleading interpretation of the clinical results, resulting in the empirical treatments in 7–13% of NS cases (2). Therefore, a precise and reliable rapid diagnosis is urgently warranted to save the neonates’ lives, especially the high-risk newborns, before the disease starts manifesting prominent clinical symptoms.

The full blood count test has gained in popularity due to its low price, easy access, and direct and multiple detections, especially in low- and middle-income countries. Although the counting of blood components like platelet, lymphocyte, and neutrophil might serve as the clinical indicators of underlying infections such as sepsis and associated immune dysfunctions (3–5). However, these parameters are usually interpreted in isolation. In recent years, the platelet-to-lymphocyte ratio (PLR), neutrophil-to-lymphocyte ratio (NLR), immature-to-total neutrophil ratio (ITR), and immature-to-mature neutrophil ratio (IMR) are considered potential markers of systemic inflammation as well as prognosis of infectious diseases (3–6). However, the current view on the diagnostic role of peripheral blood leucocyte ratios is still controversial due to conflicting results in different studies. A recent review by Russell et al. (5) based on 40 studies has shown that peripheral blood leucocyte ratio could be the most relevant clinical marker of both local and systemic infections, having most of the supporting evidence related to the diagnosis of bacterial and viral infections. Data in children are limited, especially in detecting NS. Currently, there are no established threshold values for the diagnostic application of PLR and NLR in NS.

To overcome this gap, we systematically conducted this comprehensive meta-analysis to ascertain the potential of peripheral blood leucocyte ratio in diagnosing NS.

## Methods

The 27-item PRISMA checklist was followed to improve the analytical transparency of the meta-analysis, which was registered (CRD42021247850) in PROSPERO.

### Trial Searching

The Cochrane, MEDLINE, Web of Science, and EMBASE databases were searched from the time of inception of the study to April 10th, 2021 using the keywords “immature to total neutrophil,” “neutrophil to lymphocyte,” “immature to mature neutrophil,” and “platelet to lymphocyte” to identify and include eligible studies without any language restrictions. The search was also updated using e-mail alerts and electronic delivery until December 31st, 2021. We further searched in reference lists of eligible articles for any additional ones that were not found in the initial search. Additional reports or studies were retrieved by searching the reference lists. Details of the search and selection strategy are presented in the Supplementary Appendix 1.

### Selection Criteria

Only the published studies, including the case-controlled, cross-sectional, and cohorts, evaluating the diagnostic indexes (PLR, NLR, ITR, and IMR) for NS diagnosis were considered.

Eligible studies included: (1) NS diagnosis based on the positive result in blood culture, and subjects either having suspected NS but negative blood culture, healthy, and no sepsis including both of the above formed the control group; (2) sufficient data to calculate statistically significant outcomes in terms of true-negative (TN), true-positive (TP), false-negative (FN), and false-positive (FP) values; and (3) PLR, NLR, ITR, and IMR measurements in serum samples done at the time of clinical presentations of NS before antibiotic therapies or during the inclusion of control subjects in the study.

Excluded studies were: review articles, other types of secondary literature, letters, animal studies, and/or non-comparative investigations (case reports/series), and studies with 10 or fewer participants.

### Data Extraction

Study relevant data were extracted and curated by two independent researchers that included: study authors, year of publication, country, design, predefined threshold, sepsis onset, gestational age, weight, characteristics, and outcomes (TP, TN, FP, and FN values, sensitivity and specificity of the data). A third reviewer helped resolve issues when there was a lack of consensus.

### Assessment of the Bias Risks

Any potential risk of study selection bias and applicability of diagnostic accuracies of concerned indexes were evaluated based on the QUADAS-2 criteria by RevMan v5.4 (7). Analyses were focused on four aspects of QUADAS-2 such as patient selection, clinical index tests, flow and timing, and the reference standards. The risk of study bias was graded as low, high, or unclear if (1) all answers were “yes” for a section; (2) any answer was “no”; or (3) provided information was insufficient, respectively. The applicability of the study was judged accordingly.

### Statistical Analysis

“Two-by-two” diagnostic tables were constructed based on the dichotomous data from index tests and the reference standards for all studies. To measure the sensitivity and specificity, study-specific forest plots [95% confidence intervals (CI)] were generated using the RevMan v5.4 platform.

The bivariate model was employed to determine the random effect pooled values for sensitivity and specificity, including the logit-transformed values for both study-wide comparisons. The summary receiver operating characteristic (SROC) as well as 95% confidence ellipses around the summary estimates of sensitivity and specificity were then calculated. Furthermore, *I*^2^ was calculated to assess heterogeneity (8), and a value of 25, 50, or 75%, respectively, corresponded to low, moderate, or high statistical heterogeneity. If heterogeneity among studies was recorded, meta-regression was used to identify its sources. The Deeks test and a funnel plot were exploited for the publication bias assessment (9), where *P* < 0.05 indicated significant bias. Depending on the nature of available data, the association between the quality of the study method and the results of sensitivity analysis was investigated by eliminating studies with a higher bias risk across the key domains. The above mentioned analyses were carried out using the MIDAS in STATA v15.

## Results

**FIGURE 1:**
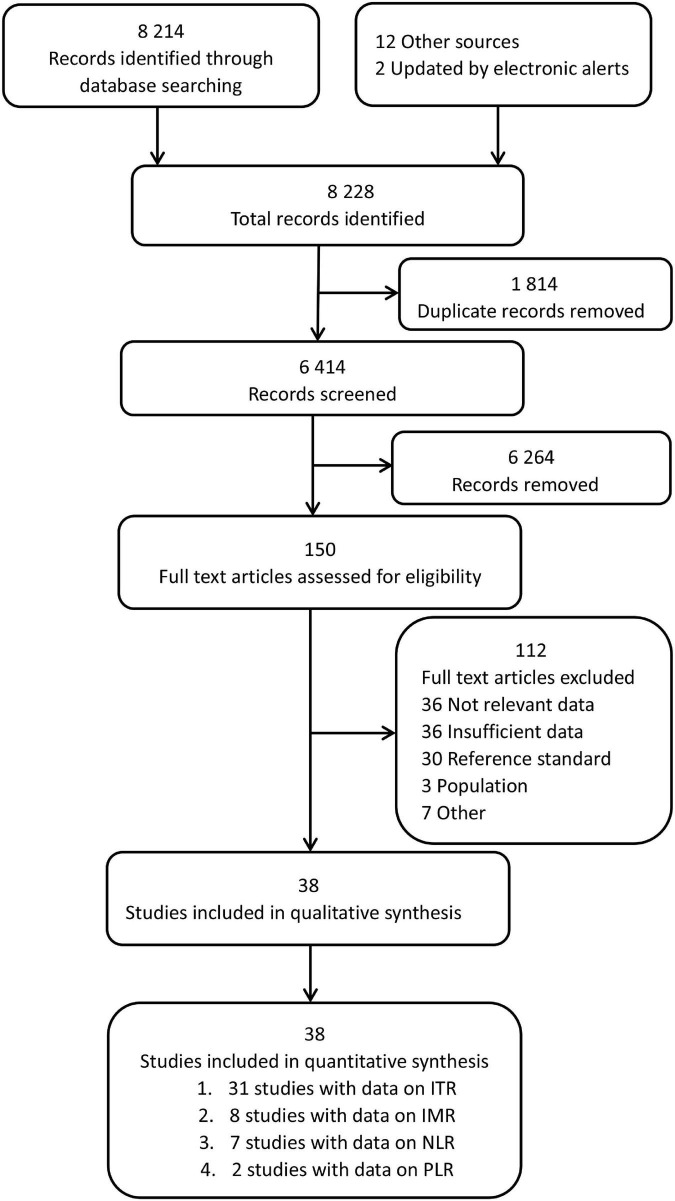
Flowchart depicting the study selection procedure.

Figure 1 illustrates the search strategy and output. Of the 8,214 identified records, 150 studies were carefully reviewed, and relevant data were retrieved. 38 studies were found eligible; additionally, 12 more studies were enrolled by searching reference lists of selected studies, and 2 studies were obtained from the index updates. A list of the excluded studies and their characteristics are provided in Supplementary Table 1.

The characteristics of enrolled studies and their respective diagnostic data are summarized in Supplementary Table 2. A total of 38 studies yielded data for the primary analysis. Of these, thirty-one studies (37 datasets, 6,221 neonates) included data on the ITR. Eight studies (10 datasets, 1,230 neonates) included data on the IMR, seven studies (7 datasets, 751 neonates) included data on the NLR, and two studies (2 datasets, 283 neonates) included data on the PLR. The present analysis was divided into several parts because studies reported diagnostic accuracy separately for the non-sepsis group, index test, and cut-off. A total of 56 datasets were analyzed as a result.

Overall, the sepsis and non-sepsis groups contained 2,830 and 5,655 participants, respectively. Region-wise, 31 (81.6%) trials enrolled participants from developing countries, while only 7 (18.4%) trials were from developed countries. In terms of sepsis onset, only early onset NS (EONS), diagnosed within 72 h of birth, patients were included in seven studies; late-onset NS (LONS), diagnosed 72 h after birth, in five studies; and the rest twenty-six studies either involved both EONS and LONS cases or didn’t mention any relevant information. Regarding the trial design, 15 cross-sectional, 8 case-control, and 16 cohort studies were included. One study was designed in two phases: cross-sectional and case-control.

### Risk-of-Bias Assessment and Publication Bias

**FIGURE 2:**
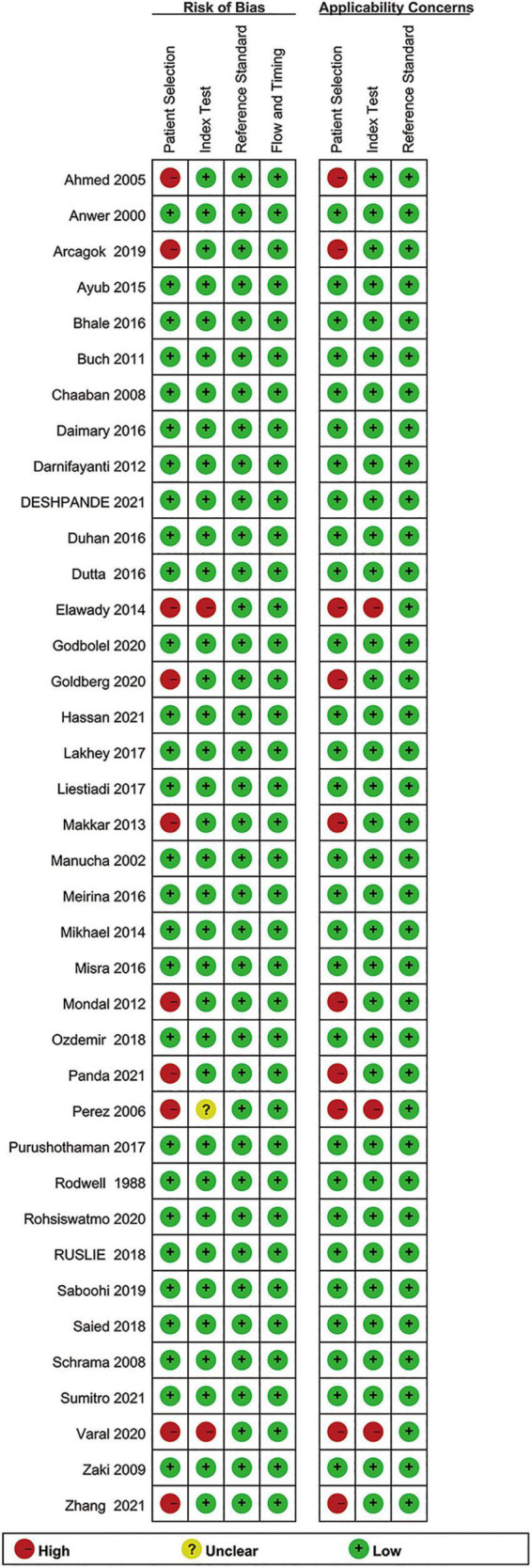
Summary of the methodological quality of the studies according to the QUADAS-2 (Quality Assessment of Diagnostic Accuracy Studies-2) criteria.

Figure 2 shows the results for the risk of bias assessment. Ten out of thirty-eight studies exhibited a high patient selection bias. Thirty-five studies showed a low bias in the index tests. All studies exhibited a low bias for flow and timing and reference standards, as well. Regarding the applicability concern, high biases were recorded only for patient selection (10 studies), and index tests (3 studies).

**FIGURE 3:**
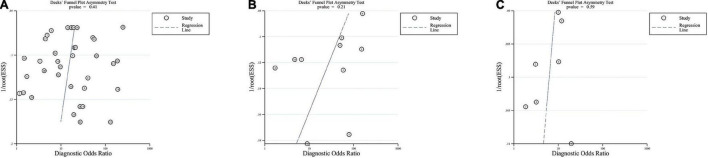
Deeks funnel plots. Funnel plots evaluating publication bias of ITR **(A)**, IMR **(B)**, and NLR **(C)**.

Figure 3 shows the results of the publication bias evaluation. Based on the *P* values of ITR, IMR, and NLR (0.41, 0.21, and 0.59, respectively), and the respective Deek’s funnel plots, we couldn’t detect any significant publication biases. The PLR could not be evaluated because only two studies included relevant data. However, the results do not ascertain the absence of any publication biases since Deeks tests lack power, particularly under the significant heterogeneous conditions.

### Findings

**FIGURE 4:**
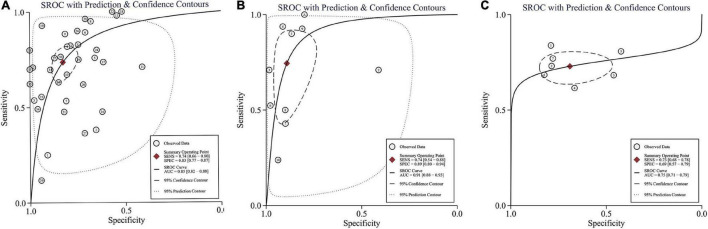
Summary of receiver operating characteristic curve for ITR **(A)**, IMR **(B)**, and NLR **(C)** with 95% confidence contour and 95% prediction contour. AUC, area under curve; SEN, sensitivity; and SPE, specificity.

**FIGURE 5:**
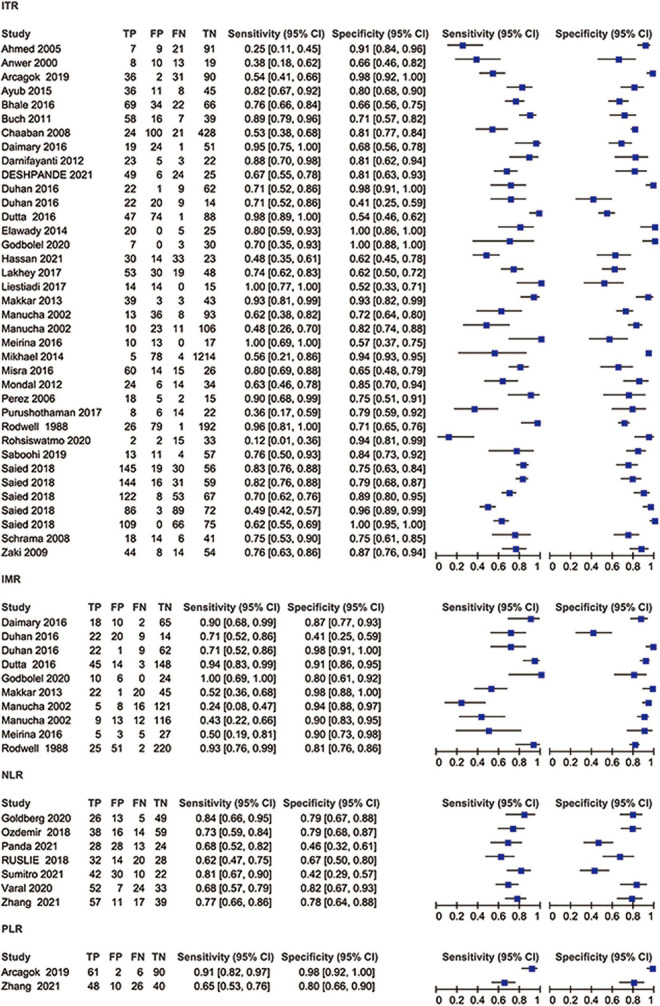
Coupled forest plots showing sensitivity and specificity of ITR, IMR, NLR, and PLR for diagnosing neonatal sepsis.

The SROC curve and forest plots are shown in Figures 4, 5.

Thirty-one studies (37 datasets) were included in the ITR test. The summary sensitivity and specificity estimates were, respectively, 0.74 (95% CI: 0.66–0.80), and 0.83 (95% CI: 0.77–0.87; Figure 4A). The area under the summary receiver operating characteristic (AUSROC) curve was 0.85 (95% CI: 0.82–0.88). A significant level of heterogeneity was recorded across the studies with the overall *I*^2^ for the bivariate model of 99% (95% CI: 94–99), and no threshold effect was evident in MIDAS. Different cut-offs might be responsible for a small proportion (0.19) of heterogeneity, and the source was identified by the meta-regression analyses. Meta-regression and subgroup analysis in Table 1 demonstrated statistically significant evidence (*P* < 0.05) of an interconnection between a few covariates (design, sepsis onset, gestational age, control group, and predefined threshold) and test accuracy. The sensitivity of cross-sectional study design was 0.78 (95% CI: 0.69–0.88), which was greater than that of the case-control [0.71 (95% CI: 0.54–0.89)], and cohort designs [0.68 (95% CI: 0.55–0.81)]. The specificity of the case-control studies [0.94 (95% CI: 0.89–0.98)] was significantly higher than that of the cross-sectional studies [0.82 (95% CI: 0.74–0.90)] and cohort studies [0.76 (95% CI: 0.66–0.86)]. The AUSROC curve was also measured, showing that the diagnostic accuracy in the case-control studies [0.93 (95% CI: 0.91–0.95)] was higher than that in the cross-sectional [0.86 (95% CI: 0.83–0.89)] and cohort [0.79 (95% CI: 0.76–0.83)] studies. In terms of onset, the sensitivity of the NS [EONS and LONS; 0.78 (95% CI: 0.72–0.84)] was significantly higher than that of EONS [0.54 (95% CI: 0.35–0.73)] and LONS [0.53 (95% CI: 0.11–0.94)], while the specificities were similar at the onset. In terms of gestational age, the sensitivity of preterm or term status [0.54 (95% CI: 0.17–0.90)] was lower than that of preterm and term status [0.75 (95% CI: 0.68–0.83)], but its specificity [0.92 (95% CI: 0.81–1.00)] was higher than that of the preterm and term status [0.83 (95% CI: 0.77–0.88)]. In terms of the control group, the sensitivity of suspected sepsis [0.75 (95% CI: 0.66–0.84)] was slightly higher than that of no sepsis [0.73 (95% CI: 0.60–0.86)] and healthy status [0.65 (95% CI: 0.40–0.90)], while the specificity of the healthy status [0.96 (95% CI: 0.92–1.00)] was significantly higher than that of no sepsis [0.90 (95% CI: 0.84–0.95)] and suspected sepsis [0.73 (95% CI: 0.66–0.80)]. Furthermore, the predefined threshold had a similar sensitivity to that of the no predefined threshold, while the diagnostic accuracy in the no predefined threshold group was higher than that in the predefined threshold group as measured by the AUSROC curve [0.86 (95% CI: 0.82–0.89)] vs. [0.84 (95% CI: 0.80–0.86)]. However, the analysis had limited power to determine the SROC curve for predesign, sepsis onset, gestational age, and control group due to the limited number of studies in each subgroup. Region, predesign and threshold analyses yielded no significant results. Pooled sensitivity [0.75 (95% CI: 0.68–0.81)] and specificity [0.82 (95% CI: 0.76–0.87)] values were similar when studies with a high-risk of bias were eliminated.

**TABLE 1:** The result of meta-regression and subgroup analysis for ITR.

Category	No. of trails	Sensitivity (95% CI)	Specificity (95% CI)	I^2^ (%)	*P*	AUSROC
**Region**						
Developed country	6	0.70 [0.50–0.90]	0.84 [0.72–0.95]	0	0.92	0.86 [0.82–0.88]
Developing country	31	0.74 [0.67–0.82]	0.83 [0.77–0.88]			0.85 [0.82–0.88]
**Predesign**						
Prospective	34	0.75 [0.68–0.82]	0.81 [0.76–0.87]	50	0.13	0.85 [0.82–0.88]
Retrospective	3	0.53 [0.21–0.84]	0.93 [0.86–1.00]			–
**Design**						
Case-control study	7	0.71 [0.54–0.89]	0.94 [0.89–0.98]	80	0.01	0.93 [0.91–0.95]
Cohort study	13	0.68 [0.55–0.81]	0.76 [0.66–0.86]	71	0.03	0.79 [0.76–0.83]
Cross-sectional study	17	0.78 [0.69–0.88]	0.82 [0.74–0.90]	0	0.49	0.86 [0.83–0.89]
**Onset**						
EONS	7	0.54 [0.35–0.73]	0.85 [0.74–0.95]	64	0.06	0.56 [0.51–0.60]
LONS	2	0.53 [0.11–0.94]	0.88 [0.71–1.00]	0	0.53	–
EONS and LONS	28	0.78 [0.72–0.84]	0.82 [0.76–0.88]	74	0.02	0.87 [0.84–0.90]
**Gestational age**						
Preterm or term	2	0.54 [0.17–0.90]	0.92 [0.81–1.00]	93	0.00	–
Preterm and term	33	0.75 [0.68–0.83]	0.83 [0.77–0.88]			0.86 [0.83–0.89]
**Control group**						
Healthy	4	0.65 [0.40–0.90]	0.96 [0.92–1.00]	79	0.01	–
No sepsis	11	0.73 [0.60–0.86]	0.90 [0.84–0.95]	65	0.06	–
Suspected sepsis	22	0.75 [0.66–0.84]	0.73 [0.66–0.80]	90	0.00	0.79 [0.75–0.82]
**Threshold predefined**						
Yes	27	0.74 [0.65–0.82]	0.79 [0.72–0.85]	68	0.04	0.84 [0.80–0.86]
No	10	0.73 [0.59–0.86]	0.90 [0.85–0.96]			0.86 [0.82–0.89]
**Threshold**						
0.2	20	0.70 [0.60–0.81]	0.81 [0.73–0.88]	31	0.24	0.82 [0.79–0.85]
Increase*	7	0.87 [0.78–0.97]	0.81 [0.69–0.94]	61	0.08	0.92 [0.89–0.94]
Other than 0.2 and Increase*	10	0.70 [0.55–0.85]	0.87 [0.79–0.95]	0	0.54	0.85 [0.82–0.88]

*EONS, early onset NS; LONS, late-onset NS; ITR, immature-to-total neutrophil ratio; and AUSROC, area under the summary receiver operating characteristic curve.*

*Normal values as defined by reference ranges of Manroe et al. (15).

Eight studies (10 datasets) were included in the IMR test. The summary sensitivity and specificity estimates were, respectively, 0.74 (95% CI: 0.54–0.88) and 0.89 (95% CI: 0.80–0.94; Figure 4B). The AUSROC curve was 0.91 (95% CI: 0.88–0.93). Studies were significantly heterogeneous. The overall *I*^2^ for the bivariate model was 96% (95% CI: 92–99), without apparent threshold effects. There was a small proportion (0.08) of heterogeneity likely due to different cut-offs. Meta-regression and sensitivity analyses were limited due to the small sample size. Pooled sensitivity [0.81 (95% CI: 0.61–0.92)], and specificity [0.90 (95% CI: 0.78–0.94)] values were similar when studies with a high-risk of bias were removed.

Seven studies (7 datasets) were included in the NLR test. The summary sensitivity and specificity estimates were 0.73 (95% CI: 0.68–0.78) and 0.69 (95% CI: 0.57–0.79; Figure 4C). The AUSROC curve was 0.75 (95% CI: 0.71–0.79). Studies were substantially heterogeneous. The overall *I*^2^ for the bivariate model was 83% (95% CI: 65–100). No threshold effect was recorded (STATA MIDAS). A small (0.03) proportion of heterogeneity was probably caused by different cutoffs. The small sample size limited the sensitivity and meta-regression analyses. Pooled sensitivity [0.71 (95% CI: 0.65–0.76)] and specificity [0.73 (95% CI: 0.62–0.81)] values were similar when studies with a high risk of bias were not included.

Two studies (2 datasets) were included in the PLR test. The summary specificity and sensitivity estimates were, respectively, 0.93 (95% CI: 0.81–1.00) and 0.81 (95% CI: 0.55–1.00). Likewise, the small sample size statistically limited the SROC curve, meta-regression, and sensitivity analyses.

## Discussion

Here, we compared the accuracy of ITR, IMR, NLR, and PLR, in predicting NS. The present review determined that all four tests were sensitive (summary sensitivity: 0.73–0.81), specific (summary specificity: 0.69–0.93), and had an AUSROC curve ranging from 0.75 to 0.91. The presence of statistically heterogeneous results in all analyses should be seriously considered during the interpretation of results. In addition, insufficient data sources did not allow us to perform direct comparisons across the test groups. Also, the number of studies mentioning the PLR test was too small to count.

In the sub-group analysis, the ITR sensitivity in NS diagnosis exhibited significant variations among the case-control, cohort, and cross-sectional studies, suggesting that the study design could be a critical modulator of the accuracy of the diagnostic trial. Some studies employed optimal cut-offs instead of predetermined ones which might have led to the overestimated diagnostic accuracy in certain instances. Also, a higher proportion of healthy neonates in the control group might have contributed to the overall improved diagnostic accuracy. The ITR cut-off of 0.20 is usually favorable for NS onset (48). However, the present results showed that the diagnostic accuracy of the ITR using Higgins et al. (8) as a reference threshold was higher compared to other cut-offs.

In 2019, a meta-analysis of five studies involving 3,320 adult subjects presenting signs of infection has reported that the AUSROC curve for the NLR is 0.72 (95% CI: 0.69–0.74) in predicting the presence of bacteremia in these patients (5). These findings are similar to those in the present study [0.75 (95% CI: 0.71–0.79)] for the identification of sepsis. In a meta-analysis by Jiang et al. (49) in 2018 that included eight studies with 7,095 adults, blood culture has been regarded as a control measure or a diagnostic criterion, showing that the summary sensitivity and specificity of NLR was 0.72 (95% CI: 0.66–0.77) and 0.59 (95% CI: 0.55–0.63) in the diagnosis of bacteremia. However, the study was restricted to the adult population and did not include a meta-analysis of the ITR, IMR, and PLR in sepsis.

### Merits and Demerits of This Review

To date, this is the first meta-analysis concerning peripheral blood leucocyte ratios in NS. In addition, blood culture was used for a confirmed episode of NS in this analysis, providing a consistent reference standard for diagnoses.

Due to the presence of considerable limitations in this study, the results should be carefully interpreted. First, a statistically small sample size, especially relating to the IMR, NLR, and PLR tests, was one of the major rate-limiting factors, as reflected in the wide ranges of 95% CI of the pooled estimates. Second, the presence of high levels of heterogeneity across studies negatively impacted the pooled estimates. Insufficient information was the third limitation. In preterm infants and those aged less than 3 days, infection was more common than in term infants and those aged more than 3 days, but the aforementioned details were rarely reported in isolation. Importantly, the index tests could have been perform differently in NS due to gram-positive/negative, or fungal pathogens. Hence, patients’ clinical characteristics and ICU-associated local microbial profile might have cumulatively affected the diagnostic index tests for NS. However, we were limited to exploring further because of the lack of essential information. Therefore, the current data are inadequate to draw any affirmative conclusions.

Finally, it wasn’t possible to ascertain that the index level determination was performed following an identical or comparable laboratory method across the studies.

Indeed, from the wide range of the cut-offs used for the ITR (ranging from 0.03 to 0.50), NLR (ranging from 1.50 to 9.40), and PLR (ranging from 57.70 to 90.84), except IMR (ranging from 0.25 to 0.30), it seemed different assay methods were implemented in different studies. It could also be possible that authors were biased in selecting threshold data that generated optimized test accuracy. Moreover, the exact timing of blood collection was not reported in a few studies, instead mentioned either “before antibiotic treatment” or “on admission.” Therefore, the specific causes for the differences in diagnostic accuracy in different laboratory procedures could not be analyzed further.

## Conclusion

The NLR may not be an appropriate indicator for the NS diagnosis. Both of ITR and IMR seem suitable for identifying NS, but limited sample size and large heterogeneity prohibit us from making a strong recommendation at this stage. Notably, there were only two studies on the PLR, which could not be evaluated. Thus, we don’t recommend ITR, IMR, NLR, or PLR as the single conclusive test for NS diagnosis at this moment. And our results should be carefully interpreted considering the patient’s physical examinations, medical history, and microbial assessment. So, persistent re-evaluation of the disease condition is highly advisable.

## Data Availability Statement

The original contributions presented in this study are included in the article/Supplementary Material, further inquiries can be directed to the corresponding author.

## Author Contributions

JJZ collected, analyzed, and interpreted the data, as well as majorly contributed to the manuscript writing. JZ served as the second reviewer for data collection, analysis, and risk of bias assessments. LZ, XY, JG, and ZL participated in data analysis, interpretation, and manuscript preparation. All authors read and approved the final version of this manuscript.

## Conflict of Interest

The authors declare that the research was conducted in the absence of any commercial or financial relationships that could be construed as a potential conflict of interest.

## Publisher’s Note

All claims expressed in this article are solely those of the authors and do not necessarily represent those of their affiliated organizations, or those of the publisher, the editors and the reviewers. Any product that may be evaluated in this article, or claim that may be made by its manufacturer, is not guaranteed or endorsed by the publisher.

## Supplementary Material

Supplementary Appendix 1 Search strategy.

Supplementary Table 1 Characteristics of excluded studies (ordered by study number).

Supplementary Table 2 Characteristics of included studies.
