# Supplementary material for: The Utility of Peripheral Blood Leucocyte Ratios as Biomarkers in Neonatal Sepsis: A Systematic Review and Meta-Analysis
Source: Front Pediatr. 2022 Jul 22;10:908362. doi: 10.3389/fped.2022.908362 (PMC9353072; doi:10.3389/fped.2022.908362)
Supplement: Supplementary Appendix 1 — Search strategy. [file Data_Sheet_1.PDF]

Search strategy

Ovid MEDLINE(R) and Epub Ahead of Print, In-Process, In-Data-Review & Other Non-Indexed Citations, Daily and Versions(R)		
1	exp Infant, Newborn/	620951
2	Premature Birth/	15052
3	(neonat$ or neo nat$).ti,ab.	274957
4	(newborn$ or new born$ or newly born$).ti,ab.	171753
5	(preterm or preterms or pre term or pre terms).ti,ab.	79611
6	(preemie$ or premie or premies).ti,ab.	180
7	(prematur$ adj3 (birth$ or born or deliver$)).ti,ab.	16322
8	(low adj3 (birthweight$ or birth weight$)).ti,ab.	36052
9	(lbw or vlbw or elbw).ti,ab.	8881
10	infan$.ti,ab.	452878
11	(baby or babies).ti,ab.	72709
12	"Intensive Care Units, Neonatal"/	15416
13	1 or 2 or 3 or 4 or 5 or 6 or 7 or 8 or 9 or 10 or 11 or 12	1091319
14	("neutrophil to lymphocyte" or "neutrophil-lymphocyte" or NLR or NLCR).ti,ab.	11175
15	("platelet to lymphocyte" or "platelet-lymphocyte" or PLR).ti,ab.	6745
16	("Immature to total neutrophil" or "Immature-total neutrophil" or "I/T" or "I:T ratio" or ITR).ti,ab.	4114
17	("immature to mature neutrophil" or "immature-mature neutrophil" or "I/M" or "I:M ratio").ti,ab.	2167
18	Biological Markers/	293978
19	biomarker$.ti,ab.	288558
20	14 or 15 or 16 or 17 or 18 or 19	512470
21	exp Sepsis/	126916
22	exp Bacterial Infections/	902982
23	exp systemic inflammatory response syndrome/	132046
24	(Sepsis or septic or Sepses) .ti,ab.	137188
25	(infection$ or Py?emia? or Septic?emia? or bacill?emia? or bacter?emia? or fung?emia?).ti,ab.	1487081
26	(blood adj2 poison$).ti,ab.	209
27	(systemic inflammatory response syndrome or SIRS).ti,ab.	7960
28	((Toxic or Endotox$ or bacter$) adj2 Shock).ti,ab.	9919
29	21 or 22 or 23 or 24 or 25 or 26 or 27 or 28	2192463
30	exp animals/ not humans.sh.	4810615
31	(13 and 20 and 29) not 30	2665
**Embase via Ovid**		
1	exp Infant/	1023646
2	Prematurity/	108262
3	(neonat$ or neo nat$).ti,ab.	359886

4	(newborn$ or new born$ or newly born$).ti,ab.	202039
5	(preterm or preterms or pre term or pre terms).ti,ab.	112440
6	(preemie$ or premie or premies).ti,ab.	285
7	(prematur$ adj3 (birth$ or born or deliver$)).ti,ab.	22751
8	(low adj3 (birthweight$ or birth weight$)).ti,ab.	45947
9	(lbw or vlbw or elbw).ti,ab.	12231
10	infan$.ti,ab.	521873
11	(baby or babies).ti,ab.	101948
12	newborn intensive care/	26743
13	1 or 2 or 3 or 4 or 5 or 6 or 7 or 8 or 9 or 10 or 11 or 12	1448789
14	("neutrophil to lymphocyte" or "neutrophil-lymphocyte" or NLR or NLCR) .ti,ab.	17668
15	("platelet to lymphocyte" or “platelet-lymphocyte” or PLR) .ti,ab.	6745
16	(“Immature to total neutrophil” or “Immature-total neutrophil” or I/T or “I:T ratio” or ITR or “IT ratio”) .ti,ab	4114
17	(“immature to mature neutrophil” or “immature-mature neutrophil” or "I/M" or “I:M ratio” or “IM ratio”) .ti,ab.	2557
18	Biological Markers/	270968
19	biomarker$.ti,ab.	449230
20	14 or 15 or 16 or 17 or 18 or 19	559631
21	exp Sepsis/	279925
22	newborn sepsis/	8514
23	sepsis.ti,ab.	157807
24	infection$.ti,ab.	1874533
25	exp Bacteremia/	52153
26	exp Fungemia/	7980
27	(bacteraemia or bacteremia).ti,ab.	40674
28	(fungaemia or fungemia).ti,ab.	2914
29	exp systemic inflammatory response syndrome/	287913
30	systemic inflammatory response syndrome.ti,ab.	7543
31	SIRS.ti,ab.	10108
32	21 or 22 or 23 or 24 or 25 or 26 or 27 or 28 or 29 or 30 or 31	2099235
33	exp animals/ not humans.sh.	27089902
34	(13 and 20 and 32) not 33	110
	**Web of Science**	
#1	**TOPIC:** (neonat* or “neo nat*” or newborn* or “new born*” or “newly born*” or preterm or preterms or “pre term” or “pre terms”or preemie* or premie* or premise or lbw or vlbw or elbw or infant or infants or infancy or baby or babies or "neonatal intensive care")	908,805
#2	**TOPIC:** (prematur* NEAR/3 (birth* or born or deliver*))	15,436
#3	**TOPIC:** (low NEAR/3 (birthweight* or “birth weight*”))	47,634
#4	#1 or #2 or #3	919,954
#5	**TOPIC:** (("neutrophil to lymphocyte" or "neutrophil-lymphocyte " or NLR or	447,480

	NLCR) OR ("platelet to lymphocyte" or “platelet-lymphocyte” or PLR) OR (“Immature to total neutrophil” or “Immature-total neutrophil” or I/T or “I:T ratio” or ITR or “IT ratio”) OR (“immature to mature neutrophil” or “immature-mature neutrophil” or "I/M" or “I:M ratio” or “IM ratio”) OR ("biological markers" or biomarker*))	
#6	**TOPIC:** (Sepsis or septic or Sepses or infection* or Py?emia? or Septic?emia? or bacill?emia? or bacter?emia? or fung?emia? or “systemic inflammatory response syndrome” or SIRS)	1,800,732
#7	**TOPIC:** (blood NEAR/2 poison*)	278
#8	**TOPIC:** ((Toxic or Endotox* or bacter*) NEAR/2 Shock)	13,098
#9	#6 or #7 or #8	1,808,185
#10	**TITLE:** ((animal* OR bovine OR cattle OR mice OR mouse OR pig OR pigs OR rabbit* OR rat OR rats))	4,818,696
#11	(#9 and #5 and #4)not #10	1,511
**The Cochrane Library**		
#1	([mh "newborn"] OR [mh "infant"] OR [mh "premature birth"] OR neonat* OR "neo nat*" OR newborn* OR "new born*" OR "newly born*" OR preterm OR preterms OR "pre term" OR "pre terms" OR preemie* OR premie* OR premise OR lbw OR vlbw OR elbw OR infant OR infants OR infancy OR baby OR babies OR "neonatal intensive care" OR (prematur* NEAR/3 (birth* OR born OR deliver*)) OR (low NEAR/3 (birthweight* OR 'birth weight*')))	92931
#2	("neutrophil to lymphocyte" or "neutrophil-lymphocyte " or NLR or NLCR) OR ("platelet to lymphocyte" or “platelet-lymphocyte” or PLR) OR ("Immature to total neutrophil" or "Immature-total neutrophil" or ITR or "I/T" or “IT ratio”) OR ("immature to mature neutrophil" or "immature-mature neutrophil" or "I/M" or "IM ratio") OR ("biological markers" or biomarker*)	164282
#3	([mh "sepsis"] OR sepsis OR septic OR sepses OR infection* OR py?emia? OR septic?emia? OR bacill?emia? OR bacter?emia? OR fung?emia? OR [mh "systemic inflammatory response syndrome"] OR “systemic inflammatory response syndrome” OR sirs OR (blood NEAR/2 poison*) OR ((toxic OR endotox* OR bacter*) NEAR/2 shock))	135611
#4	#1 and #2 and #3	4032
