# Supplementary material for: The Utility of Peripheral Blood Leucocyte Ratios as Biomarkers in Neonatal Sepsis: A Systematic Review and Meta-Analysis
Source: Front Pediatr. 2022 Jul 22;10:908362. doi: 10.3389/fped.2022.908362 (PMC9353072; doi:10.3389/fped.2022.908362)
Supplement: Supplementary Table 1 — Characteristics of excluded studies (ordered by study number). [file Table_1.DOCX]

| Table S1 Characteristics of excluded studies [ordered by study ID] | |
| --- | --- |
| Study | Reason for exclusion |
| Adly 2014 | Not relevant - No data in paper |
| Alasmi 2011 | Insufficient data. Conference abstract. Authors' contact details not provided. |
| Alexejew 1990 | Insufficient data. Abstract only. Authors' contact details not provided. |
| Ang 1990 | Insufficient data. Abstract only. Authors' contact details not provided. |
| Aulia 2003 | Insufficient data. Conference abstract. Authors' contact details not provided |
| Beltempo 2018 | Not relevant - No data in paper |
| Bender 2008 | Reference standard not eligible |
| Bentlin 2007 | Reference standard not eligible |
| Berger 1995 | Insufficient data in paper to determine 2 x 2 table |
| Bhandari 2008 | Reference standard not eligible |
| Bhargava 2011 | Insufficient data. Conference abstract. Authors' contact details not provided |
| Blommendah 2002 | Insufficient data in paper to determine 2 x 2 table |
| Bohuon 1999 | Insufficient data. Title only. Authors' contact details not provided |
| Can 2018 | Reference standard not eligible |
| Chen 2021 | Not relevant - No data in paper |
| Christensen 1981 | Not relevant - No data in paper |
| Chwals 1994 | Population not eligible-Average age was 47 days |
| Dapaah-Siakwan 2016 | Not relevant - No data in paper |
| Doughty 2020 | Not relevant - No data in paper |
| Dujic-Bilusic 2014 | Insufficient data. Title only. Authors' contact details not provided |
| El Gawhary 2016 | Not relevant - No data in paper |
| El-Mashad 2016 | Not relevant - No data in paper |
| El-Sonbaty 2016 | Not relevant - No data in paper |
| Fang 2015 | Index test not eligible. CD64 expressed on neutrophils, monocytes, and lymphocytes |
| Felicitus 2015 | Insufficient data. Conference abstract. Authors' contact details not provided |
| Franz 1999 | Population not eligible- Infected infants |
| Gerdes 1987 | Reference standard not eligible |
| Ghosh 2001 | Data not eligible to populate 2×2 table. |
| Ghrahani 2019 | Reference standard not eligible |
| Golding 2020 | Not relevant - No data in paper |
| Gonzalez 2003 | Not relevant - No data in paper |
| Gupta 2017 | Insufficient data. Conference abstract. Authors' contact details not provided |
| Hamie 2018 | Population not eligible- Infected infants |
| Hancioglu 1991 | Insufficient data. Abstract only. Authors' contact details not provided |
| Hansen 2005 | Not relevant - No data in paper |
| Hashem 2020 | Reference standard not eligible |
| Heredia 2021 | Insufficient data. Conference abstract. Authors' contact details not provided |
| Hornik 2012 | Reference standard not eligible |
| Janota 2000 | Insufficient data. Abstract only. Authors' contact details not provided. |
| Janota 2001 | Reference standard not eligible |
| Jialin 2013 | Insufficient data. Abstract only. Authors' contact details not provided. |
| Karabulut 2021 | Reference standard not eligible |
| Karabulut 2020 | Data duplication |
| Kazanasmaz 2019 | Insufficient data. Abstract only. We contacted the authors but received no reply. |
| Khair 2012 | Data not eligible to populate 2×2 table. |
| Khattab 2018 | Reference standard not eligible |
| Kordek 2011 | Reference standard not eligible |
| Krauel 1987 | Insufficient data. Abstract only. Authors' contact details not provided. |
| Krediet 1992 | Population not eligible- Infected infants |
| Kudawla 2008 | Not relevant – No NLR, PLR, I/T or I/M |
| Laborada 2003 | Reference standard not eligible |
| Li 2020 | Reference standard not eligible |
| Liu 2014 | Not relevant - No data in paper |
| MacQueen 2016 | Not relevant - No neonatal sepsis |
| Mahale 2010 | Reference standard not eligible |
| Mahmoud 2009 | Insufficient data. Abstract only. Authors' contact details not provided. |
| Makhoul 2006 | Not relevant - No data in paper |
| McMaster 2009 | Population not eligible- Their median age was 52 weeks |
| Morales 2015 | Insufficient data. Conference abstract. Authors' contact details not provided |
| Murphy 2012 | Not relevant - No data in paper |
| Nabi 2019 | Insufficient data. Abstract only. We contacted the authors but received no reply. |
| Narasimha 2011 | Reference standard not eligible |
| Neunhoeffer 2015 | Reference standard not eligible |
| Newman 2014 | Insufficient data in paper to determine 2 x 2 table |
| O'Carroll 2010 | Insufficient data. Conference abstract. Authors' contact details not provided |
| Omran 2018 | Reference standard not eligible |
| Ongun 2020 | Not relevant - No data in paper |
| Orbak 2003 | Not relevant - No data in paper |
| Orlikowsky 2004 | Reference standard not eligible |
| Pauli 1999 | Not relevant - No data in paper |
| Pecheva 1995 | Insufficient data. Conference abstract. Authors' contact details not provided |
| Perez Solis 2006 | Reference standard not eligible |
| Raimondi 2010 | Insufficient data. Conference abstract. Authors' contact details not provided |
| Selimovic 2008 | Insufficient data. Abstract only. We contacted the authors, but the letter was returned. |
| Resch 2015 | Not relevant - No data in paper |
| Rite Gracia 2003 | Reference standard not eligible |
| Rodwell 1993 | Not relevant - No data in paper |
| Rohadi 2020 | Not relevant - No data in paper |
| Rosenfeld 2019 | Data not eligible to populate 2×2 table |
| Russell 1992 | Reference standard not eligible |
| Saldir 2015 | Reference standard not eligible |
| Samra 2019 | Reference standard not eligible |
| Sarkar 2015 | No relevant data |
| Schlapbach 2013 | Reference standard not eligible |
| Shams 2017 | Reference standard not eligible |
| Shao 2005 | No relevant data |
| Shapiro 1984 | Insufficient data. Conference abstract. Authors' contact details not provided |
| Silveira 1999 | No relevant data |
| Slavikova 1989 | Insufficient data. Title only. Authors' contact details not provided |
| Smulian 1997 | No relevant data |
| Speer 1985 | Insufficient data. Abstract only. Authors' contact details not provided |
| Stempniewicz 1995 | Insufficient data. Abstract only. Authors' contact details not provided |
| Streimish 2014 | Insufficient data in paper to determine 2 x 2 table |
| Taskin 2020 | No relevant data |
| Tegtmeyer 1992 | No relevant data |
| Terrin 2011 | Reference standard not eligible |
| Tunc 2015 | Reference standard not eligible |
| Uras 2010 | Reference standard not eligible |
| Van 2018 | No relevant data |
| Varsha 2003 | Insufficient data. Abstract only. Authors' contact details not provided |
| Walliullah 2009 | Insufficient data. Abstract only. Authors' contact details not provided |
| Waliullah 2010 | Insufficient data. Abstract only. Authors' contact details not provided |
| Wang 2017 | Insufficient data in paper to determine 2 x 2 table |
| Wang 2020 | No relevant data |
| Weitkamp 2000 | No relevant data |
| Wilar 2019 | Reference standard not eligible |
| Wilar 2016 | Reference standard not eligible |
| Wojcik-Zygadlo 1998 | Insufficient data. Abstract only. Authors' contact details not provided |
| Wojsyk-Banasza 2002 | Insufficient data. Abstract only. Authors' contact details not provided |
| Yadav 2005 | No relevant data |
| Zelada 1989 | Insufficient data. Abstract only. Authors' contact details not provided |
| Zhang 2018 | No relevant data |
