# Supplementary material for: The Utility of Peripheral Blood Leucocyte Ratios as Biomarkers in Neonatal Sepsis: A Systematic Review and Meta-Analysis
Source: Front Pediatr. 2022 Jul 22;10:908362. doi: 10.3389/fped.2022.908362 (PMC9353072; doi:10.3389/fped.2022.908362)
Supplement: Supplementary Table 2 — Characteristics of included studies. [file Table_2.docx]

| **Table S2 Characteristics of Included Studies** | | | | | | | | | | | | | | |
| --- | --- | --- | --- | --- | --- | --- | --- | --- | --- | --- | --- | --- | --- | --- |
| Source | Country | Recruitment | Study population | | | | Ratio | Threshold  Predefined | TP | FP | FN | TN | Sensitivity  (95% CI) | Specificity  (95% CI) |
|  |  |  | Sepsis onset | GA | BW | NS VS Control group |  |  |  |  |  |  |  |  |
| Ahmed ^[10]^  2005 | Switzerland | Cross-sectional | EONS  LONS | Preterm  Term | NBW  LBW | Proven sepsis  VS  Healthy neonate | ITR | 0.20（Yes） | 7 | 9 | 21 | 91 | 0.25[0.11,0.45] | 0.91[0.84,0.96] |
| Anwer^[11]^  2000 | Pakistan | Prospective  Cohort | EONS  LONS | Preterm  Term | NBW  LBW | Proven sepsis  VS  Suspected sepsis | ITR | 0.20（Yes） | 8 | 10 | 13 | 19 | 0.38[0.18,0.62] | 0.66[0.46,0.82] |
| Arcagok^[12]^  2019 | Turkey | Retrospective  Case-control | EONS | Term | NBW | Proven sepsis  VS  Healthy neonate | ITR  PLR | 0.22（No）  57.70（No） | 36  61 | 2  2 | 31  6 | 90  90 | 0.54[0.41,0.66]  0.91[0.82,0.97] | 0.98[0.92,1.00]  0.98[0.92,1.00] |
| Ayub^[13]^  2015 | Pakistan | Cross-sectional | EONS  LONS | Preterm  Term | NBW  LBW | Proven sepsis  VS  Suspected sepsis | ITR | 0.20（Yes） | 36 | 11 | 8 | 45 | 0.82[0.67,0.92] | 0.80[0.68,0.90] |
| Bhale^[14]^  2016 | India | Cross-sectional | EONS  LONS | Preterm  Term | NBW  LBW | Proven sepsis  VS  Suspected sepsis | ITR | 0.20（Yes） | 69 | 34 | 22 | 66 | 0.76[0.66,0.84] | 0.66[0.56,0.75] |
| Buch^[15]^  2011 | India | Prospective Cohort | EONS  LONS | Preterm  Term | NBW  LBW | Proven sepsis  VS  Suspected sepsis | ITR | 0.20（Yes） | 58 | 16 | 7 | 39 | 0.89[0.79,0.96] | 0.71[0.57,0.82] |
| Chaaban^[16]^  2008 | United States | Prospective Cohort | EONS  LONS | Preterm | NR | Proven sepsis  VS  Suspected sepsis | ITR | 0.16（Yes） | 24 | 100 | 21 | 428 | 0.53[0.38,0.68] | 0.81[0.77,0.84] |
| Daimary^l[17]^  2016 | India | Prospective Cohort | EONS  LONS | Preterm  Term | NR | Proven sepsis  VS  Suspected sepsis | ITR  IMR | Increase*（Yes）  0.30（Yes） | 19  18 | 24  10 | 1  2 | 51  65 | 0.95[0.75,1.00]  0.90[0.68,0.99] | 0.68[0.56,0.78]  0.87[0.77,0.93] |
| Darnifayanti ^[18]^  2012 | Indonesia | Cross-sectional | EONS  LONS | Preterm  Term | NBW  LBW | Proven sepsis  VS  Suspected sepsis | ITR | 0.20（Yes） | 23 | 5 | 3 | 22 | 0.88[0.70,0.98] | 0.81[0.62,0.94] |
| DESHPANDE^[19]^  2021 | India | Prospective Cohort | EONS  LONS | Preterm  Term | NBW  LBW | Proven sepsis  VS  Suspected sepsis | ITR | 0.20（Yes） | 49 | 6 | 24 | 25 | 0.67[0.55,0.78] | 0.81[0.63,0.93] |
| Duhan^[20]^  2016 | India | Cross-sectional Case-control | EONS  LONS | Preterm  Term | NBW  LBW | Proven sepsis  VS  Suspected sepsis  Proven sepsis  VS  Healthy neonate | ITR  IMR  ITR  IMR | Increase*（Yes）  0.30（Yes）  Increase*（Yes）  0.30（Yes） | 22  22  22  22 | 20  20  1  1 | 9  9  9  9 | 14  14  62  62 | 0.71[0.52,0.86]  0.71[0.52,0.86]  0.71[0.52,0.86] 0.71[0.52,0.86] | 0.41[0.25,0.59] 0.41[0.25,0.59]  0.98[0.91,1.00] 0.98[0.91,1.00] |
| Dutta ^[21]^  2016 | India | Cross-sectional | EONS  LONS | Preterm  Term | NBW  LBW | Proven sepsis  VS  Suspected sepsis | ITR  IMR | 0.20（Yes）  0.30（Yes） | 47  45 | 74  14 | 1  3 | 88  148 | 0.98[0.89,1.00] 0.94[0.83,0.99] | 0.54[0.46,0.62] 0.91[0.86,0.95] |
| Elawady^[22]^  2014 | Egypt | Prospective  case-control | EONS  LONS | Preterm  Term | NBW  LBW | Proven sepsis  VS  No sepsis | ITR | 0.20（No） | 20 | 0 | 5 | 25 | 0.80[0.59,0.93] | 1.00[0.86,1.00] |
| Godbole^[23]^  2020 | India | Cross-sectional | EONS  LONS | Preterm  Term | NR | Proven sepsis  VS  Suspected sepsis | ITR  IMR | 0.20（Yes）  0.30（Yes） | 7  10 | 0  6 | 3  0 | 30  24 | 0.70[0.35,0.93]  1.00[0.69,1.00] | 1.00[0.88,1.00]  0.80[0.61,0.92] |
| Goldberg^[24]^  2020 | Israel | Retrospective  case-control | LONS | Preterm  Term | NBW  LBW | Proven sepsis  VS  No sepsis | NLR | 1.50（No） | 26 | 13 | 5 | 49 | 0.84[0.66,0.95] | 0.79[0.67,0.88] |
| Hassan ^[25]^  2021 | India | Prospective Cohort | EONS | Preterm  Term | NBWI  LBW | Proven sepsis  VS  Suspected sepsis | ITR | 0.20（Yes） | 30 | 14 | 33 | 23 | 0.48[0.35,0.61] | 0.62[0.45,0.78] |
| Lakhey^[26]^  2017 | Nepal | Cross-sectional | EONS  LONS | Preterm  Term | NBW  LBW | Proven sepsis  VS  Suspected sepsis | ITR | 0.20（Yes） | 53 | 30 | 19 | 48 | 0.74[0.62,0.83] | 0.62[0.50,0.72] |
| Liestiadi^[27]^  2017 | Indonesia | Cross-sectional | EONS  LONS | Preterm  Term | NBW  LBW | Proven sepsis  VS  Suspected sepsis | ITR | 0.12（Yes） | 14 | 14 | 0 | 15 | 1.00[0.77,1.00] | 0.52[0.33,0.71] |
| Makkar^[28]^  2013 | India | Prospective  Case-control | EONS  LONS | Preterm  Term | NBW  LBW | Proven sepsis  VS  Healthy neonate | ITR  IMR | Increase*（Yes）  0.30（Yes） | 39  22 | 3  1 | 3  20 | 43  45 | 0.93[0.81,0.99] 0.52[0.36,0.68] | 0.93[0.82,0.99] 0.98[0.88,1.00] |
| Manucha^[29]^  2002 | India | Prospective  Cohort | EONS | Preterm  Term | NBW  LBW | Proven sepsis  VS  Suspected sepsis | ITR  IMR  ITR  IMR | 0.14（No）  0.25（No）  0.16（Yes）  0.30（Yes） | 13  9  10  5 | 36  13  23  8 | 8  12  11  16 | 93  116  106  121 | 0.62[0.38,0.82] 0.43[0.22,0.66]  0.48[0.26,0.70] 0.24[0.08,0.47] | 0.72[0.64,0.80] 0.90[0.83,0.95]  0.82[0.74,0.88] 0.94[0.88,0.97] |
| Meirina^[30]^  2016 | Indonesia | Cross-sectional | EONS  LONS | Preterm  Term | NBW  LBW | Proven sepsis  VS  Suspected sepsis | ITR  IMR | Increase*（Yes）  0.30（Yes） | 10  5 | 13  3 | 0  5 | 17  27 | 1.00[0.69,1.00] 0.50[0.19,0.81] | 0.57[0.37,0.75] 0.90[0.73,0.98] |
| Mikhael^[31]^  2014 | United States | Retrospective  Cohort | EONS | Preterm  Term | NBW  LBW | Proven sepsis  VS  No sepsis | ITR | Increase*（Yes） | 5 | 78 | 4 | 1214 | 0.56[0.21,0.86] | 0.94[0.93,0.95] |
| Misra^[32]^  2016 | India | Prospective  Cohort | EONS  LONS | NR | NR | Proven sepsis  VS  Suspected sepsis | ITR | 0.20（Yes） | 60 | 14 | 15 | 26 | 0.80[0.69,0.88] | 0.65[0.48,0.79] |
| Mondal^[33]^  2012. | India | Prospective  Case-control | EONS  LONS | Preterm  Term | NBW  LBW | Proven sepsis  VS  No sepsis | ITR | 0.20（Yes） | 24 | 6 | 14 | 34 | 0.63[0.46,0.78] | 0.85[0.70,0.94] |
| Ozdemir^[34]^  2018 | Turkey | Prospective  Cohort | LONS | Preterm | LBW | Proven sepsis  VS  Suspected sepsis | NLR | 1.77（No） | 38 | 16 | 14 | 59 | 0.73[0.59,0.84] | 0.79[0.68,0.87] |
| Panda^[35]^  2021 | India | Retrospective  Case control | EONS  LONS | Preterm  Term | NBWI  LBW | Proven sepsis  VS  No sepsis | NLR | 1.70（No） | 28 | 28 | 13 | 24 | 0.68[0.52,0.82] | 0.46[0.32,0.61] |
| Perez^[36]^  2006 | Spain | Prospective  Case-control | LONS | Preterm  Term | NBW  LBW | Proven sepsis  VS  No sepsis | ITR | 0.03（No） | 18 | 5 | 2 | 15 | 0.90[0.68,0.99] | 0.75[0.51,0.91] |
| Purushothaman^[37]^  2017 | India | Prospective  Cohort | EONS | NR | NR | Proven sepsis  VS  Suspected sepsis | ITR | 0.20（Yes） | 8 | 8 | 14 | 20 | 0.36[0.17,0.59] | 0.79[0.59,0.92] |
| Rodwell ^[38]^  1988 | Australia | Prospective  Cohort | EONS  LONS | Preterm  Term | NBW  LBW | Proven sepsis  VS  Suspected sepsis | ITR  IMR | Increase*（Yes）  0.30（Yes） | 26  25 | 79  51 | 1  2 | 192  220 | 0.96[0.81,1.00] 0.93[0.76,0.99] | 0.71[0.65,0.76] 0.81[0.76,0.86] |
| Rohsiswatmo^[39]^  2020 | Indonesia | Cross-sectional | LONS | Preterm  Term | NBW  LBW | Proven sepsis  VS  Suspected sepsis | ITR | 0.20（Yes） | 2 | 2 | 15 | 33 | 0.12[0.01,0.36] | 0.94[0.81,0.99] |
| RUSLIE ^[40]^  2018 | Indonesia | Retrospective  Cohort | EONS  LONS | Preterm  Term | NBW  LBW | Proven sepsis  VS  Suspected sepsis | NLR | 9.40（No） | 32 | 14 | 20 | 28 | 0.62[0.47,0.75] | 0.67[0.50,0.80] |
| Saboohi^[41]^  2019 | Pakistan | Cross-sectional | EONS | Preterm  Term | NBW  LBW | Proven sepsis  VS  Suspected sepsis | ITR | 0.20（Yes） | 13 | 11 | 4 | 57 | 0.76[0.50,0.93] | 0.84[0.73,0.92] |
| Saied^[42]^  2018 | Egypt | Cross-sectional | EONS  LONS | Preterm  Term | NBW  LBW | Proven sepsis  VS  No sepsis | ITR | 0.15（No）  0.20（No）  0.25（No）  0.30（No）  0.50（No） | 145  144  122  86  109 | 19  16  8  3  0 | 30  31  53  89  66 | 56  59  67  72  75 | 0.83[0.76,0.88] 0.82[0.76,0.88]  0.70[0.62,0.76] 0.49[0.42,0.57]  0.62[0.55,0.69] | 0.75[0.63,0.84] 0.79[0.68,0.87]  0.89[0.80,0.95] 0.96[0.89,0.99]  1.00[0.95,1.00] |
| Schrama^[43]^  2008 | The Netherlands | Prospective  Cohort | EONS  LONS | Preterm  Term | NBW  LBW | Proven sepsis  VS  No sepsis | ITR | 0.20（Yes） | 18 | 14 | 6 | 41 | 0.75[0.53,0.90] | 0.75[0.61,0.85] |
| Sumitro^[44]^  2021 | Indonesia | Cross-sectional | EONS  LONS | Preterm  Term | NBW  LBW | Proven sepsis  VS  Suspected sepsis | NLR | 2.12（No） | 42 | 30 | 10 | 22 | 0.81[0.67,0.90] | 0.42[0.29,0.57] |
| Varal^[45]^  2020 | Turkey | Retrospective Cohort | LONS | Preterm | LBW | Proven sepsis  VS  No sepsis | NLR | 1.57（No） | 52 | 7 | 24 | 33 | 0.68[0.57,0.79] | 0.82[0.67,0.93] |
| Zakil^[46]^  2009 | Egypt | Prospective  Cohort | EONS  LONS | Preterm  Term | NBW  LBW | Proven sepsis  VS  No sepsis | ITR | 0.20（No） | 44 | 8 | 14 | 54 | 0.76[0.63,0.86] | 0.87[0.76,0.94] |
| Zhang ^[47]^  2021 | China | Cross-sectional | EONS | Preterm  Term | NBW  LBW | Proven sepsis  VS  No sepsis | NLR  PLR | 3.17（No）  90.84（No） | 57  48 | 11  10 | 17  26 | 39  40 | 0.77[0.66,0.86]  0.65[0.53,0.76] | 0.78[0.64,0.88] 0.80[0.66,0.90] |
| Abbreviations: BW, birth weight; NS, neonatal sepsis; EONS, early-onset NS; LONS, late-onset NS; ITR, immature-to-total neutrophil ratio; NLR, neutrophil-to-lymphocyte ratio; PLR , platelet-to-lymphocyte ratio ; IMR, immature-to-mature neutrophil ratio; GA, gestational age; NR, not reported; LBW, Low birth weight; NBW, normal birth weight;*Normal Values as defined by reference ranges of Manroe, et al^[15]^. | | | | | | | | | | | | | | |

| **Table 2 The result of meta-regression and subgroup analysis for ITR** | | | | | | |
| --- | --- | --- | --- | --- | --- | --- |
| **Category** | **NO.of Trails** | **Sensitivity(95%CI)** | **Specificity(95%CI)** | **I^2^(%)** | **P** | **AUSROC** |
| **Region** |  |  |  |  |  |  |
| Developed country | 6 | 0.70 [0.50 - 0.90] | 0.84 [0.72 - 0.95] | 0 | 0.92 | 0.86 [0.82 - 0.88] |
| Developing country | 31 | 0.74 [0.67 - 0.82] | 0.83 [0.77 - 0.88] |  |  | 0.85 [0.82 - 0.88] |
| **Predesign** |  |  |  |  |  |  |
| Prospective | 34 | 0.75 [0.68 - 0.82] | 0.81 [0.76 - 0.87] | 50 | 0.13 | 0.85 [0.82 - 0.88] |
| Retrospective | 3 | 0.53 [0.21 - 0.84] | 0.93 [0.86 - 1.00] |  |  | - |
| **Design** |  |  |  |  |  |  |
| Case-control study | 7 | 0.71 [0.54 - 0.89] | 0.94 [0.89 - 0.98] | 80 | 0.01 | 0.93 [0.91 - 0.95] |
| Cohort study | 13 | 0.68 [0.55 - 0.81] | 0.76 [0.66 - 0.86] | 71 | 0.03 | 0.79 [0.76 - 0.83] |
| Cross-sectional study | 17 | 0.78 [0.69 - 0.88] | 0.82 [0.74 - 0.90] | 0 | 0.49 | 0.86 [0.83 - 0.89] |
| **Onset** |  |  |  |  |  |  |
| EONS | 7 | 0.54 [0.35 - 0.73] | 0.85 [0.74 - 0.95] | 64 | 0.06 | 0.56 [0.51 - 0.60] |
| LONS | 2 | 0.53 [0.11 - 0.94] | 0.88 [0.71 - 1.00] | 0 | 0.53 | - |
| EONS and LONS | 28 | 0.78 [0.72 - 0.84] | 0.82 [0.76 - 0.88] | 74 | 0.02 | 0.87 [0.84 - 0.90] |
| **Gestational age** |  |  |  |  |  |  |
| Preterm or Term | 2 | 0.54 [0.17 - 0.90] | 0.92 [0.81 - 1.00] | 93 | 0.00 | - |
| Preterm and Term | 33 | 0.75 [0.68 - 0.83] | 0.83 [0.77 - 0.88] |  |  | 0.86 [0.83 - 0.89] |
| **Control group** |  |  |  |  |  |  |
| Healthy | 4 | 0.65 [0.40 - 0.90] | 0.96 [0.92 - 1.00] | 79 | 0.01 | - |
| No sepsis | 11 | 0.73 [0.60 - 0.86] | 0.90 [0.84 - 0.95] | 65 | 0.06 | - |
| Suspected sepsis | 22 | 0.75 [0.66 - 0.84] | 0.73 [0.66 - 0.80] | 90 | 0.00 | 0.79 [0.75 - 0.82] |
| **Threshold Predefined** | |  |  |  |  |  |
| Yes | 27 | 0.74 [0.65 - 0.82] | 0.79 [0.72 - 0.85] | 68 | 0.04 | 0.84 [0.80 - 0.86] |
| No | 10 | 0.73 [0.59 - 0.86] | 0.90 [0.85 - 0.96] |  |  | 0.86 [0.82 - 0.89] |
| **Threshold** |  |  |  |  |  |  |
| 0.2 | 20 | 0.70 [0.60 - 0.81] | 0.81 [0.73 - 0.88] | 31 | 0.24 | 0.82 [0.79 - 0.85] |
| Increase* | 7 | 0.87 [0.78 - 0.97] | 0.81 [0.69 - 0.94] | 61 | 0.08 | 0.92 [0.89 - 0.94] |
| Other than 0.2 and Increase* | 10 | 0.70 [0.55 - 0.85] | 0.87 [0.79 - 0.95] | 0 | 0.54 | 0.85 [0.82 - 0.88] |
| Abbreviations: EONS, early-onset NS; LONS, late-onset NS; ITR, immature-to-total neutrophil ratio; *Normal Values as defined by reference ranges of Manroe, et al^[15]^; AUSROC, area under the summary receiver operating characteristic curve. | | | | | | |
